# Pamphlets

**Published:** 1854-05

**Authors:** 


*Pamphlets.*—We have received from the author, a pamphlet on "Epidemics and Sanitary Reform," by Dr. M. M. Rodgers, of Rochester. We have not had time for its perusal.

A Report to the Indiana State Medical Society on Asiatic Cholera, by George Sutton, M. D., gives a full account of the various epidemics, the regions of country invaded, and the practice most usual in its management.

Prof. R. J. Breakenridge sends us his Address to the Graduates of the Kentucky School of Medicine — Session of 1853–4. It adds another to the very able introductories and addresses which have covered our table for the past few months.

"Outlines of the Principles and Practice adopted in the Orthopœdic Institution of Brooklyn," is the title of a pamphlet by Drs. Bauer and Barthelmess, the physicians of that institution. H.

